# TMEM132: an ancient architecture of cohesin and immunoglobulin domains define a new family of neural adhesion molecules

**DOI:** 10.1093/bioinformatics/btx689

**Published:** 2017-10-27

**Authors:** Luis Sanchez-Pulido, Chris P Ponting

**Affiliations:** Medical Research Council Human Genetics Unit, IGMM, University of Edinburgh, Edinburgh, UK

## Abstract

**Summary:**

The molecular functions of TMEM132 genes remain poorly understood and under-investigated despite their mutations associated with non-syndromic hearing loss, panic disorder and cancer. Here we show the full domain architecture of human TMEM132 family proteins solved using in-depth sequence and structural analysis. We reveal them to be five previously unappreciated cell adhesion molecules whose domain architecture has an early holozoan origin prior to the emergence of choanoflagellates and metazoa. The extra-cellular portions of TMEM132 proteins contain five conserved domains including three tandem immunoglobulin domains, and a cohesin domain homologue, the first such domain found in animals. These findings strongly predict a cellular adhesion function for TMEM132 family, connecting the extracellular medium with the intracellular actin cytoskeleton.

**Supplementary information:**

Supplementary data are available at *Bioinformatics* online.

## 1 Introduction

Many genes remain experimentally under-investigated not because they are functionally less important but because their discovery came relatively late (Pandey *et al.*, 2014). Our ignorance of aspects of basic biology and disease thus is perpetuated by the serendipitous order by which genes were first characterized. The need to experimentally determine proteins’ normal molecular functions, and their molecular dysfunction in disease, becomes more critical when sequence variants within functionally enigmatic genes are robustly associated with Mendelian or complex disease, or with cancer progression. Determining the molecular functions of such poorly characterized genes is all the more difficult when their protein sequences lack recognizable domains, because these otherwise can reliably provide structural and functional information through homology-based inference. Here, we shed much light on the previously unknown domain structure and functions of the 5 proteins of the human TMEM132 family (TMEM132A, B, C, D and E).

These are genes in which variants are enriched for individuals with hearing loss, panic disorder or cancer. A homozygous missense mutation in human *TMEM132E* (Arg420Gln) was confirmed using a zebrafish model to cause autosomal-recessive nonsyndromic hearing loss (Li *et al.*, 2015). Common variants within the *TMEM132E* gene are associated with insomnia symptoms (Lane *et al.*, 2017); common and rare variants near *TMEM132D* gene are robustly associated with panic disorder (Erhardt *et al.*, 2011, 2012; Hodgson *et al.*, 2016; Howe *et al.*, 2016; Inoue *et al.*, 2015; Quast *et al.*, 2012; Shimada-Sugimoto *et al.*, 2016; Wang *et al.*, 2016); and variants near TMEM132B are associated with excessive daytime sleepiness (Lane *et al.*, 2017). In healthy individuals, some of the *TMEM132D* non-coding variants exhibit higher anxiety scores and larger volumetric estimates of the amygdala and hippocampus, key neural structures associated with fear and anxiety (Haaker *et al.*, 2014). Furthermore, in cattle the TMEM132D locus appears to have undergone a selective sweep during domestication (Qanbari *et al.*, 2014), and in the mouse, anterior cingulate cortex TMEM132D expression correlates with anxiety-related behaviour (Erhardt *et al.*, 2011). Finally, mutations in *TMEM132D* are unusually frequent in small-cell lung cancer (Iwakawa *et al.*, 2015; Peifer *et al.*, 2012; Rudin *et al.*, 2012) and in pancreatic cancer (Forbes *et al.*, 2015). Disease mutations near, or functions of, *TMEM132A* or *C* have yet to be identified, although TMEM132A is thought to promote neuronal cell survival by regulating stress-related genes (Oh-hashi *et al.*, 2003, 2006, 2010, 2012, 2015). Functions of the single fruit fly (CG14446) or nematode (Y71H2AM.10) orthologous genes are yet to be described.

**Fig. 1:**
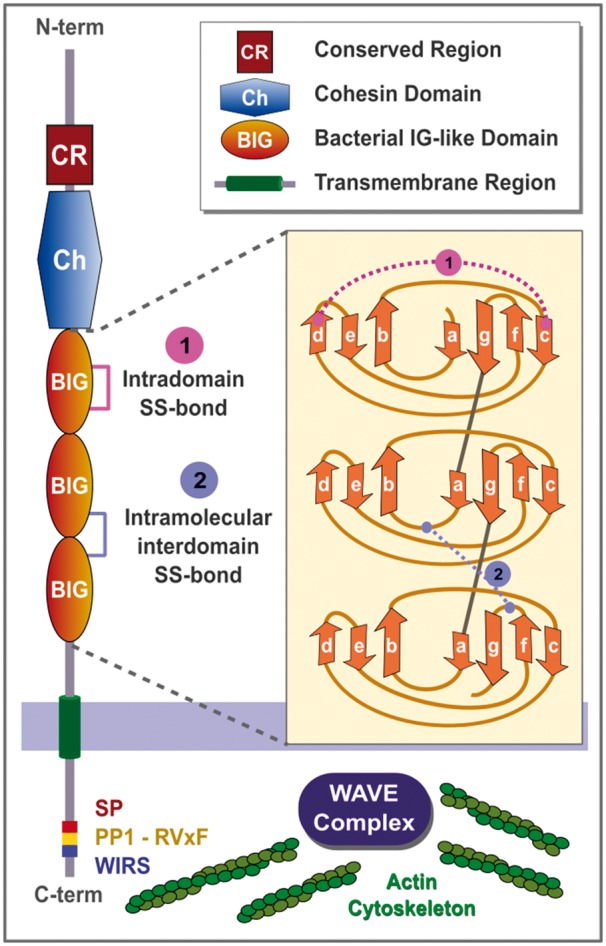
Domain architecture and common features in the TMEM132 family. Schematic representation of conserved domains present in TMEM132 family members: a conserved region (CR; shown in red; see Supplementary Fig. S6) precedes a cohesin domain (blue; see Supplementary Fig. S5), and three adjacent BIG domains (orange; Supplementary Figs S3 and S4). The predicted intradomain and interdomain disulphide bridges of these BIG domains are indicated in the right panel (magenta and violet lines, respectively). The seven beta-strands, forming part of the immunoglobulin-like core of BIG domains, are labelled a-g following an established convention (Bork *et al.*, 1994) (Supplementary Fig. S7). Evolutionarily conserved TMEM132 intracellular motifs putatively related with the control of actin cytoskeletal dynamics are: a putative serine phosphorylation motif (SP), a phosphatase-1 (PP1) interaction motif (RVxF) (Hendrickx *et al.*, 2009; Heroes *et al.*, 2013), and a WIRS (WAVE regulatory complex interacting receptor sequence) cytoplasmic motif (Chen *et al.*, 2014)

All five human paralogues encode cell-surface molecules expressed in the brain (Oh-Hashi, 2010; Nomoto *et al.*, 2003; Walser *et al.*, 2011; Uhlen *et al.*, 2010) whose amino acid sequences contain no distinguishable features other than their N-terminal signal peptides and C-terminal proximal transmembrane sequences (Nomoto *et al.*, 2003; Oh-hashi *et al.*, 2010, 2012), with intracellular C-terminal Ser/Thr phosphatase-1 (PP1) docking (Hendrickx *et al.*, 2009; Heroes *et al.*, 2013) and WIRS (WAVE regulatory complex interacting receptor sequence) cytoplasmic motifs (Fig. 1 and Supplementary Fig. S1) (Chen *et al.*, 2014). The latter is consistent with the reported co-localization of TMEM132D with actin filaments (Walser *et al.*, 2011).

Cell–cell junctions in the central nervous system (CNS) are maintained by a variety of transmembrane proteins that signal and physically link between the cytoskeletons of adjacent cells. Many such proteins (e.g. CNTN, LRIG, NCAM, SEMA3 and SIGLEC families) contain one or more immunoglobulin (IG) superfamily domains (Rougon and Hobert, 2003). Here we identify TMEM132 molecules as novel IG domain containing proteins of the CNS.

## 2 Results and discussion

### 2.1 Protein sequence analysis: three tandem immunoglobulin domains

We initiated our analyses by performing a JackHMMER iterative search (Finn *et al.*, 2015) starting from the human TMEM132A protein sequence of the UniRef50 database (Wu, 2006). Whilst characterising the TMEM132 family we identified full-length homologous proteins across essentially all of the animal kingdom, including nematodes (*Caenorhabditis elegans*) and hexapods (*Drosophila melanogaster*). As input for our analysis we used a full-length multiple sequence alignment generated with T-Coffee (Notredame *et al.*, 2000). Using extensive profile-to-sequence and profile-to-profile comparison analyses (Finn *et al.*, 2015; Söding *et al.*, 2005) we identified a repeated pattern of conserved amino-acids in the region lying between positions 400 and 767 for human TMEM132A, corresponding approximately to the conserved region used to define the family in Pfam (Family TMEM132, accession: PF16070) (Punta *et al.*, 2012). This region is conserved among animals and some premetazoan proteins that are additionally rich in cadherin domains (Supplementary Fig. S2) (Abedin and King, 2008; Nichols *et al.*, 2012). Profile-versus-sequence and profile-versus-profile comparisons of this conserved repeated pattern allowed the identification of three consecutive repeated regions, each of which independently yielded statistically significant E-values of sequence similarity with the same fold, the bacterial immunoglobulin-like (BIG) domain (Mei *et al.*, 2015; Ptak *et al.*, 2014). HHpred searches against the PDB70 profile database. (Söding *et al.*, 2005) using TMEM132 repeats 1, 2 and 3 as input (corresponding to amino acids 400–491, 495–630 and 641–767 of human TMEM132A) detected the BIG domain from *Leptospira interrogans* (PDB ID: 2mh4), for example, with E-values of 0.031, 0.018 and 0.022, respectively. Moreover in all three HHpred results, in support of the first match, the next most statistically significant matches corresponded to additional members of the immunoglobulin superfamily (Supplementary Figs S3 and S4). The PDB70 database contains profile hidden Markov models (HMMs) for representative sequences, clustered to 70% maximum pairwise sequence identity to reduce redundancy, drawn from the PDB database (Söding *et al.*, 2005).

BIG domains are widely distributed among bacteria, archaea and eukaryotes (Pfam family Big_2, accession: PF02368) (Punta *et al.*, 2012). This domain adopts a beta-sandwich fold composed of nine strands organized in three sheets. Two of these sheets (composed of seven strands) contribute the immunoglobulin-like core of BIG domains. These seven strands are labelled ‘a’ to ‘g’ in Figure 1 and Supplementary Figure S3, following an established convention in the immunoglobulin fold (Bork *et al.*, 1994). BIG domains have been described with diverse functions, usually relating to matrix, protein–ligand, or protein–protein interactions and are mainly extracellular (Mei *et al.*, 2015; Ptak *et al.*, 2014).

Close 3D proximity and evolutionary conservation of four cysteines allow us to identify two putative disulphide bridges, one that is internal to BIG1 and another that is inter-domain between BIG2 and BIG3 domains (Fig. 1; Supplementary Figs S3, S4 and S7). Disulphide bridges are commonly found in different IG folds and contribute to their structural stability (Bork *et al.*, 1994).

### 2.2 First animal cohesin domain

Identification of the three tandem BIG domains, then allowed our analyses to be focused on the TMEM132 family N-terminal region taking advantage of iterative profile-versus-sequence searches against the UniRef50 protein sequence database (Wu, 2006). These resulted in the identification of two additional domains, each of which is present not only in animals but also among more diverse eukaryotes, including members of the Coherin family in choanoflagellates and sponges (Nichols *et al.*, 2012). The domain preceding the BIG domains was discovered as the first cohesin homology domain in vertebrates (HHpred E-value < 5 × 10^−3^) (Supplementary Fig. S5). Cohesin domains are found widely in prokaryotes but, in eukaryotes, were previously thought to be restricted to choanoflagellate and sponge proteins (Pfam accession: PF00963) (Abedin and King, 2008; Nichols *et al.*, 2012; Peer *et al.*, 2009). These are not to be confused with the cohesin complex that regulates the separation of sister chromatids. Rather, cohesin domains are highly specialized protein-protein interaction modules that bind dockerin domains together forming the core that glues together the Cellulosome complex, a multi-enzymatic complex present in cellulolytic bacteria specialized in degrading cellulose (Adams *et al.*, 2008; Artzi *et al.*, 2017; Bras *et al.*, 2016; Pinheiro *et al.*, 2008; Tavares *et al.*, 1997). Bacterial cohesin-dockerin rupture forces (>120 pN) are among the highest ever reported for a receptor-ligand system (Nash *et al.*, 2016; Stahl *et al.*, 2012). It is unclear whether the TMEM132 cohesin domain mediates such a strong interaction, in part because dockerin domain homologues are not detectable in vertebrate proteins.

### 2.3 TMEM132 domain architecture is ancient

The conserved region (corresponding to amino acids 127–239 of human TMEM132A) preceding the cohesin domain in TMEM132 is also evident in choanoflagellate and sponge proteins (HMMER E-value < 0.005) (Supplementary Fig. S6). Strikingly, despite each of the five TMEM132 domains (Fig. 1) being identified independently in these choanoflagellate and sponge proteins, all five are both present and in the identical order in the three cadherin protein families—lefftyrins, coherins and hedglings—that were contained in the last common ancestor of choanoflagellates and metazoans (Abedin and King, 2008; Nichols *et al.*, 2012) (Supplementary Fig. S2). The TMEM132 domain architecture is thus ancient, preceding the emergence of early metazoans, and a repeated constituent of ancient cadherin domain-containing proteins with roles connecting the actin cytoskeleton with neighbouring cells and the extracellular matrix (Brieher and Yap, 2013; Ratheesh and Yap, 2012).

### 2.4 Disease and biological relevance

Eleven proteins are currently known to contain missense mutations within IG domains associated with 23 different disorders (Letunic *et al.*, 2015). To these now can be added a twelfth, TMEM132E, whose R420Q missense mutation, mapped to its second BIG domain (Supplementary Fig. S1), has been validated using a zebrafish model to cause autosomal-recessive nonsyndromic hearing loss (Li *et al.*, 2015). A TMEM132B nonsynonymous variant that replaces a serine conserved in TMEM132B-E in a putative phosphorylation motif has been associated with intra-cranial aneurysm (Farlow *et al.*, 2015) although this variant also occurs rarely (frequency 1 × 10^−4^) in the general population (Lek *et al.*, 2016). This lies adjacent to the Ser/Thr PP1 docking (Hendrickx *et al.*, 2009; Heroes *et al.*, 2013) and WIRS cytoplasmic motifs (Fig. 1), the latter which is found in a variety of neurological and other proteins including protocadherins (Chen *et al.*, 2014). The newly identified domains, and conserved domain architecture, of TMEM132 proteins now should facilitate detailed experimental investigation of these proteins' domain and molecular functions and how these are modulated by sequence variants.

## 3 Conclusion

Their ancient ancestry and their associations with neurological disease suggest that TMEM132 genes have been undeserving of their relative obscurity. Our identification of these proteins as CNS-expressed IG domain superfamily adhesion molecules now places them in a more appropriate perspective as a putative key connection between the extracellular matrix and the actin-based cell cytoskeleton, with major roles in regulating changes in neuronal cell morphology, motility and migration. These findings should precipitate more detailed experimental and structural characterization of the TMEM132 family, and assist in formulating hypotheses concerning the cellular mechanisms by which sequence variants in these genes contribute to neurological disease.

## Funding

This work has been supported by the Medical Research Council UK.

*Conflict of Interest*: none declared.

## Supplementary Material

Supplementary Data
