# Supplementary figures and images for: TMEM132: an ancient architecture of cohesin and immunoglobulin domains define a new family of neural adhesion molecules
Source: Bioinformatics. 2017 Oct 27;34(5):721–4. doi: 10.1093/bioinformatics/btx689 (PMC6030884; doi:10.1093/bioinformatics/btx689)

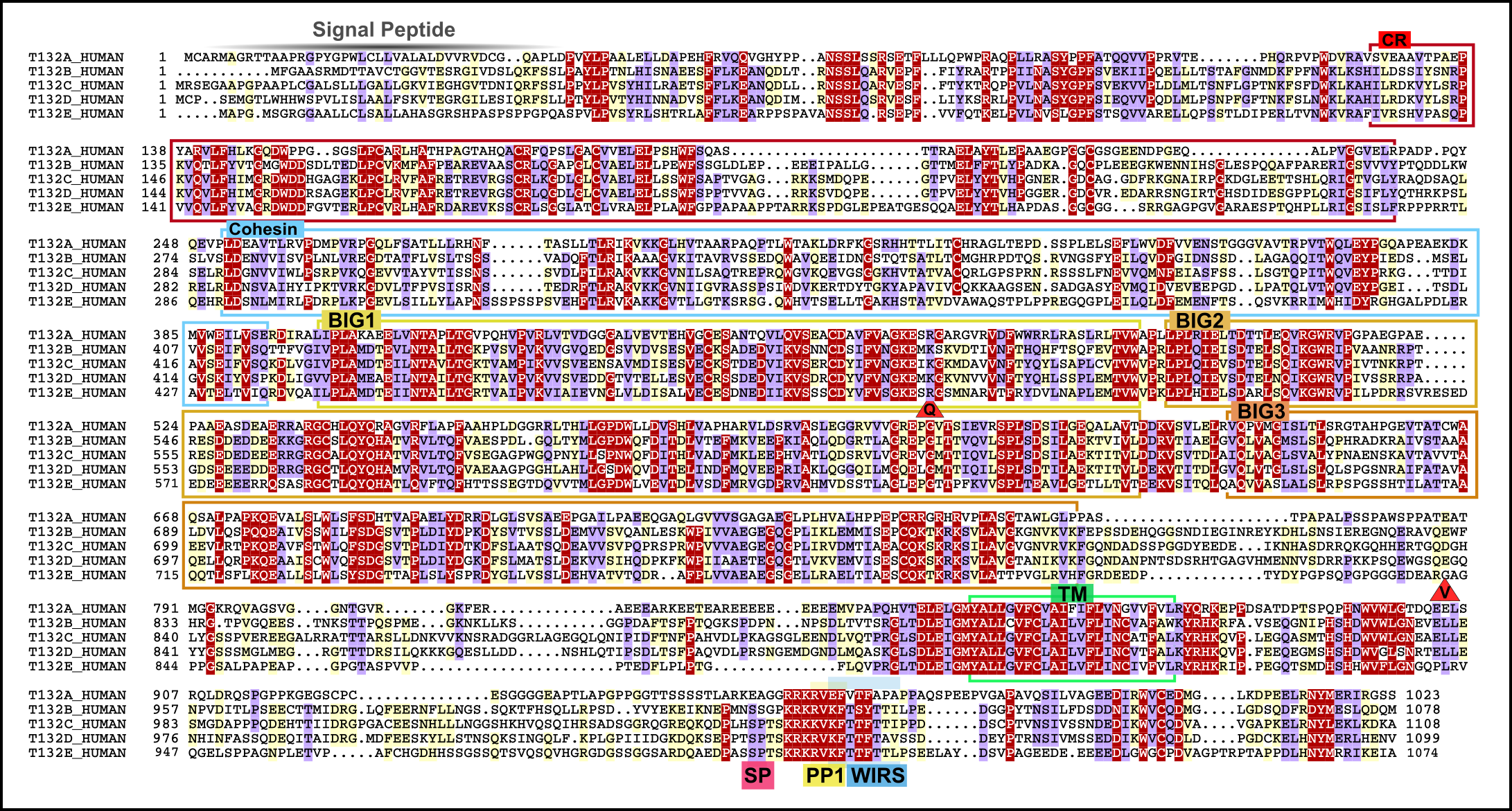

Supplement: Supplementary Data [file btx689_supp.zip › btx689-suppl_data/S1.png]

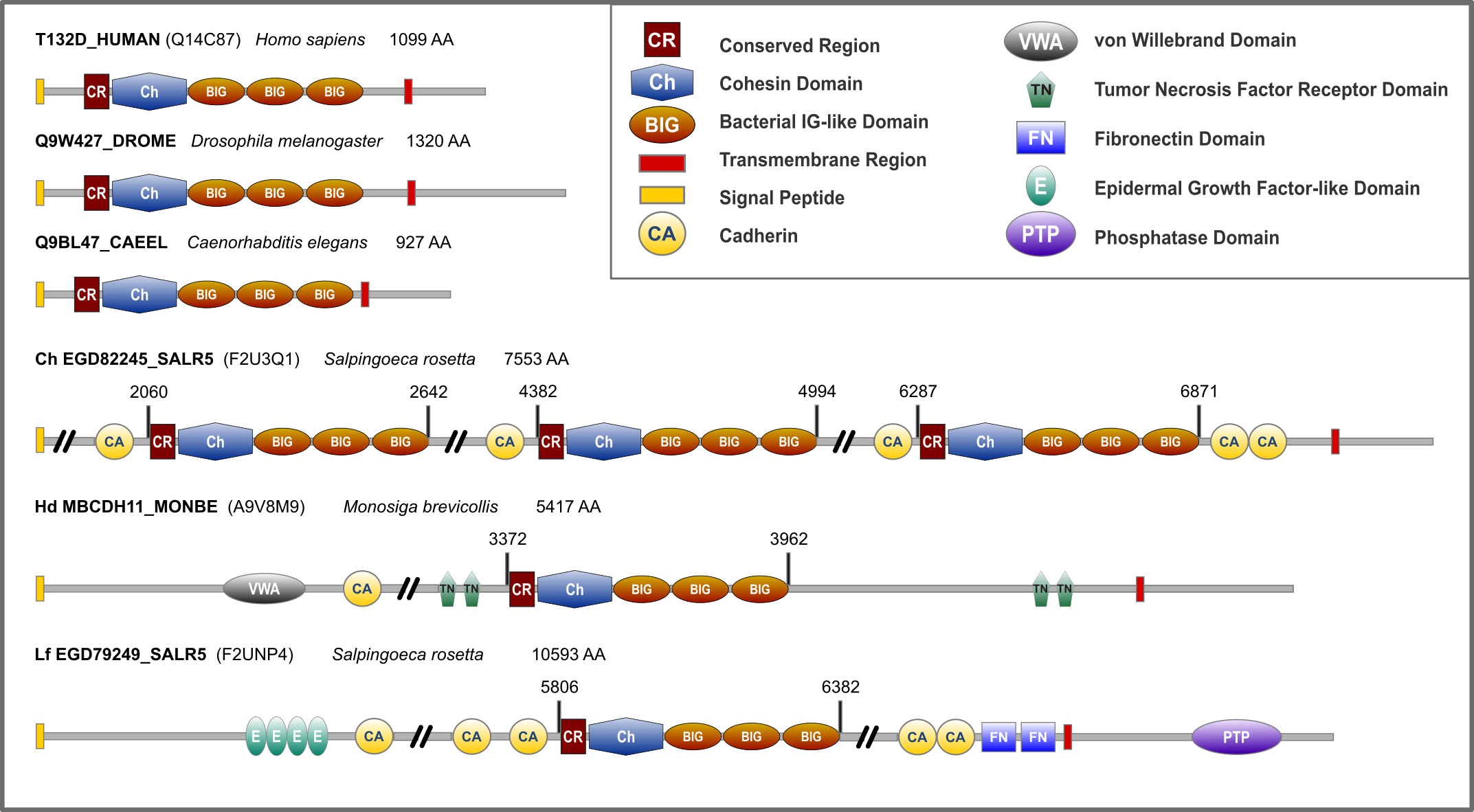

Supplement: Supplementary Data [file btx689_supp.zip › btx689-suppl_data/S2.png]

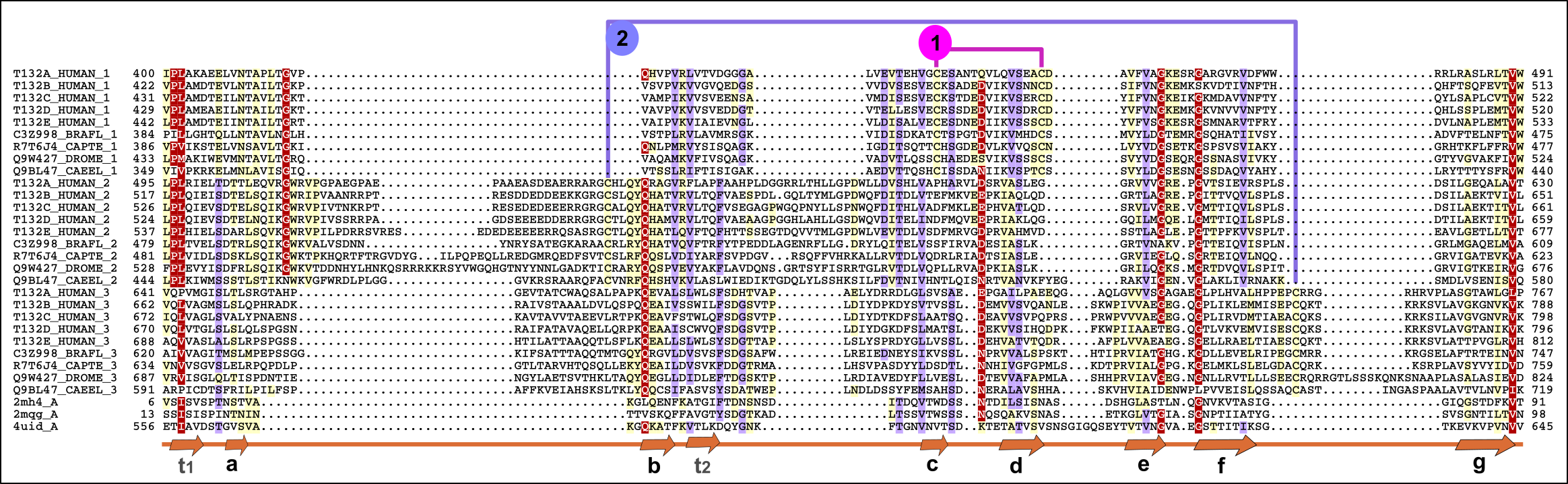

Supplement: Supplementary Data [file btx689_supp.zip › btx689-suppl_data/S3.png]

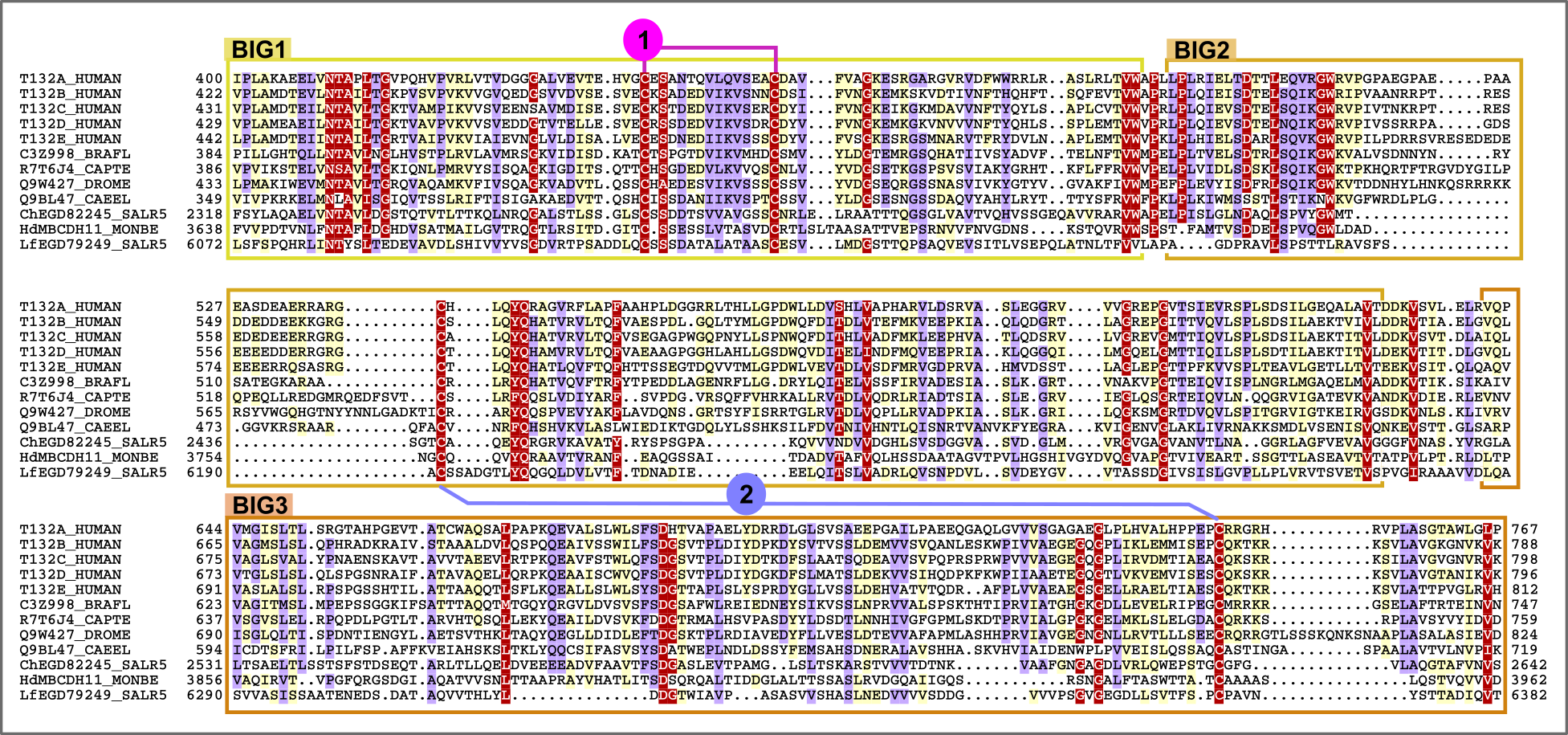

Supplement: Supplementary Data [file btx689_supp.zip › btx689-suppl_data/S4.png]

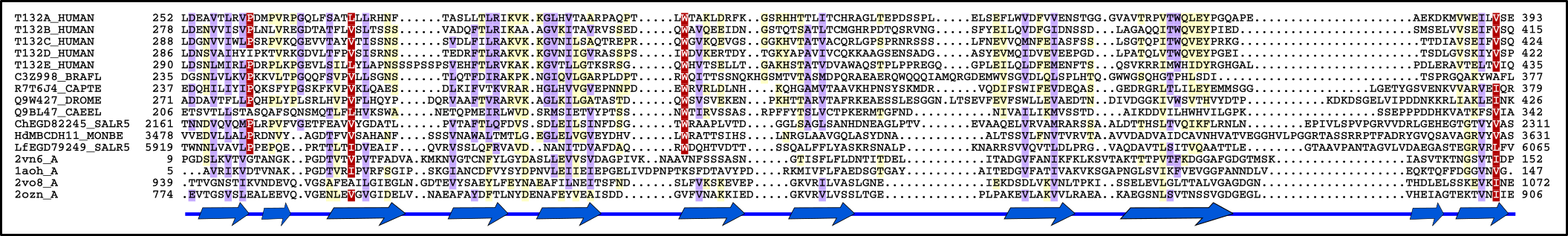

Supplement: Supplementary Data [file btx689_supp.zip › btx689-suppl_data/S5.png]

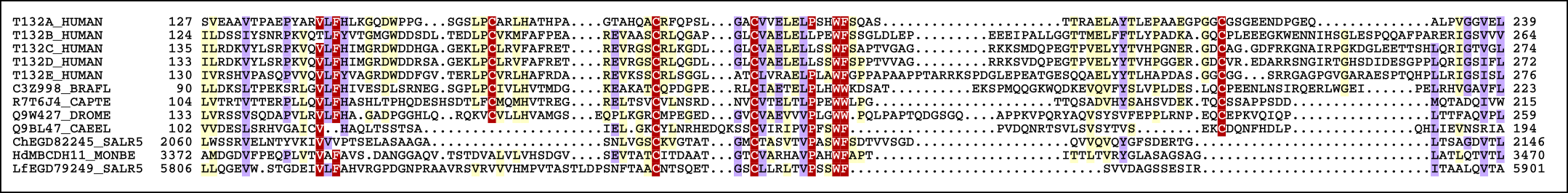

Supplement: Supplementary Data [file btx689_supp.zip › btx689-suppl_data/S6.png]

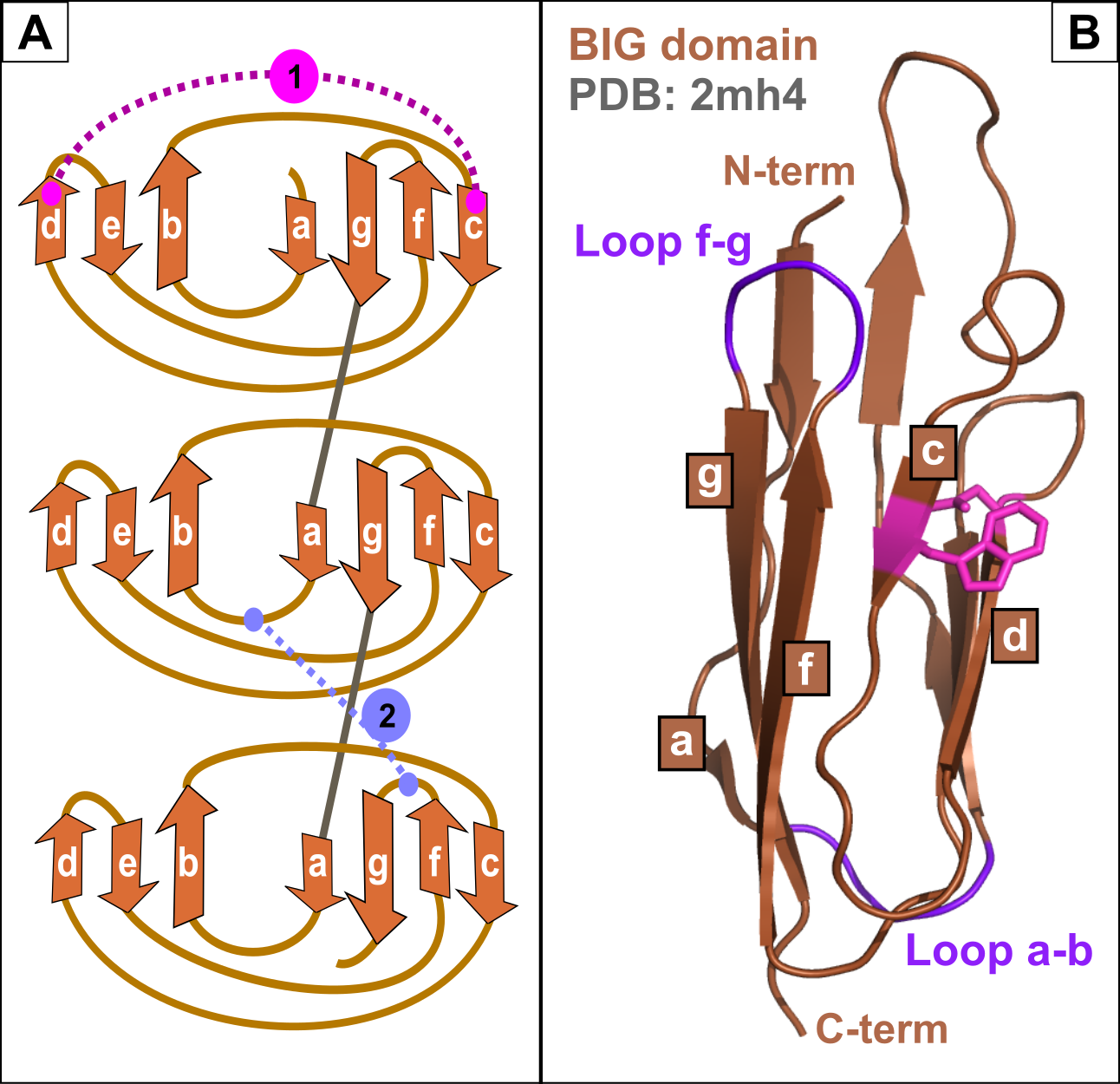

Supplement: Supplementary Data [file btx689_supp.zip › btx689-suppl_data/S7.png]
