# Supplementary material for: TMEM132: an ancient architecture of cohesin and immunoglobulin domains define a new family of neural adhesion molecules
Source: Bioinformatics. 2017 Oct 27;34(5):721–4. doi: 10.1093/bioinformatics/btx689 (PMC6030884; doi:10.1093/bioinformatics/btx689)
Supplement: Supplementary Data [file btx689_supp.zip › btx689-suppl_data/SanchezPulidoSupplementalRev.doc]

**Supplemental Figures**

**Figure S1**

**Representative full sequence alignment of human TMEM132 family members.** Coloured rectangles indicate conserved domains in the TMEM132 family: a conserved region preceding the cohesin domain (shown in red; Supplemental Figure S6); a predicted cohesin domain (in blue; Supplemental Figure S5); three consecutive BIG domains (orange; Supplemental Figures S3 and S4); and, a predicted transmembrane region (green). TMEM132 intracellular motifs putatively related with the control of actin cytoskeletal dynamics are: a putative serine phosphorylation motif (SP), a phosphatase-1 (PP1) interaction motif (RVxF), and a WIRS (WAVE regulatory complex interacting receptor sequence) cytoplasmic motif (Hendrickx *et al.*, 2009; Heroes *et al.*, 2012; Chen *et al.*, 2014) . The position of a missense mutation in human TMEM132E (R420Q) that is causal of autosomal-recessive nonsyndromic hearing loss (Li *et al.*, 2015) is indicated. A missense mutation in human TMEM132D (E838V) that is identified in the COSMIC database as occurring frequently in pancreatic cancer is also labelled (Forbes *et al.*, 2015). The amino acid colouring scheme indicates the average BLOSUM62 score (correlated to amino acid conservation) in each alignment column: red (greater than 3), violet (between 3 and 1.5) and light yellow (between 1.5 and 0.5). Analysis of known human and mouse phosphosites in the PhosphoSitePlus database (https://www.phosphosite.org/) for the TMEM132 family, shows experimental evidence of phosphorylation at positions for TMEM132E (human S915 and mouse S914) and TMEM132C (human S1046); these are aligned and highlighted in magenta.

**Figure S2**

**Domain Architecture for TMEM132 family and related premetazoan proteins.** Domain architectures of the TMEM132 family and related premetazoan proteins, according to this analysis (Supplemental Figures S3, S4, S5 and S6), Pfam, and SMART domain databases (Punta *et al.*, 2012; Letunic *et al.*, 2015). The Ch, Hd, and Lf prefixes identify members of Coherin, Hedgling, and Lefftyrin families, respectively (Abedin and King, 2008; Nichols *et al.*, 2012).

**Figure S3**

**Representative multiple sequence alignment of the BIG domains in TMEM132 family.** Predicted intradomain and interdomain disulphide bridges are indicated using magenta and violet lines (1 and 2), respectively. The seven beta-strands, part of the immunoglobulin-like core of the BIG domains, are labelled a-to-g following the established naming convention (Bork *et al.*, 1994). Two beta strands that form part of an additional beta sheet which is characteristic of BIG domains are labelled t1 and t2 (Mei *et al.*, 2015; Ptak *et al.*, 2014;). The colouring scheme indicates average BLOSUM62 score (correlated to amino acid conservation) in each alignment column: red (greater than 3), violet (between 3 and 1.5) and light yellow (between 1.5 and 0.5). The sequences are named with their Uniprot identifiers.

**Figure S4**

**Representative multiple sequence alignment of three consecutive BIG domains.** Predicted intradomain and interdomain disulphide bridges are indicated using magenta and violet lines (1 and 2), respectively. The colouring scheme indicates average BLOSUM62 score (correlated to amino acid conservation) in each alignment column: red (greater than 3), violet (between 3 and 1.5) and light yellow (between 1.5 and 0.5). Other abbreviations: see Supplemental Figure S2 legend.

**Figure S5**

**Representative multiple sequence alignment of the Cohesin domain.** The colouring scheme indicates average BLOSUM62 score (correlated to amino acid conservation) in each alignment column: red (greater than 1.5), violet (between 1.5 and 0.7) and light yellow (between 0.7 and 0.2). Consensus secondary structure of the Cohesin family is shown below the alignment in blue (Pinheiro *et al.*, 2008; Tavares *et al.*, 1997; Adams *et al.*, 2008). Other abbreviations: see Supplemental Figure S2 legend.

**Figure S6**

**Representative multiple sequence alignment of the N-terminal conserved region.** The conserved region (CR) likely extends further N-terminally. The CR limits shown here are conservative, and contain the regions of greatest sequence similarity across divergent homologues. The colouring scheme indicates average BLOSUM62 score (correlated to amino acid conservation) in each alignment column: red (greater than 3), violet (between 3 and 1.5) and light yellow (between 1.5 and 0.5). Other abbreviations: see Supplemental Figure S2 legend.

**Figure S7**

**Disulphide-bridge prediction.** Prediction of both disulphide-bridges (labelled 1 and 2) were based on cysteine conservation and likely structural proximity. In panel **A**, topological diagram of three consecutive immunoglobulin-like domains shown in Figure 1. In panel **B**, putative 3D localisation of TMEM132 conserved cysteines mapped to a known BIG domain structure (PDB: 2mh4) using the alignment shown in Supplemental Figure S3: **I)** For the putative intradomain disulphide-bridge (labelled 1), cysteines are localised in adjacent strands that are in close proximity (beta strands c and d); and, **II)** For the putative interdomain disulphide-bridge (labelled 2), cysteines are localised on opposite ends of the BIG domain: loop a-b lies close to the BIG domain C-terminus, and loop g-f lies close to the BIG domain N-terminus. Consequently, these two loops (a-b and g-f) are likely to be in close proximity within a tandem BIG domain pair between repeats 2 and 3.
